# Assessment of Physical Inactivity and Barriers to Physical Activity Among the General Population in the Jazan Region of Saudi Arabia: A Cross-Sectional Study

**DOI:** 10.7759/cureus.72295

**Published:** 2024-10-24

**Authors:** Ali Sabei, Mohammed Dahhas

**Affiliations:** 1 General Practice, Jazan University, Jazan, SAU; 2 General Practice, Primary Care Center in Sahaleel, Jazan, SAU

**Keywords:** environmental factors, health-related factors, international physical activity questionnaire, jazan region, noncommunicable diseases, obesity, physical activity, saudi arabia, saudi vision 2030, sociodemographic factors

## Abstract

Background

Lack of physical activity is a growing public health concern in Saudi Arabia, contributing to an increase in noncommunicable diseases. However, there is limited research on physical activity patterns and their associated factors in the Jazan region.

Objective

This study aimed to assess physical activity levels and identify sociodemographic, health-related, and environmental factors associated with physical activity among individuals in the Jazan region of Saudi Arabia.

Methods

This cross-sectional survey was conducted in Jazan. Physical activity was assessed using the abridged Arabic form of the International Physical Activity Questionnaire (IPAQ). Data on sociodemographic and health-related characteristics were collected using a self-administered questionnaire. Mann-Whitney U and Kruskal-Wallis tests were used to compare physical activity levels among groups, while Spearman's rank correlation was employed to investigate the relationships between continuous variables and physical activity.

Results

A total of 387 people completed the survey, 39.0% of whom were female and 61.0% were male. The participants' average age was 34.0 ± 10.1 years, and 98.4% were Saudi nationals. The study found that 42.1% of participants did not engage in high-intensity physical activity, while 39.8% did not engage in moderate-intensity activity. A strong association was found between income level, obesity, diabetes status, and high-intensity physical activity (p < 0.05). Proximity to recreational facilities was associated with moderate-intensity exercise (p = 0.05). Male participants reported walking more frequently than female participants (p = 0.033). Additionally, people with diabetes walked more often than those without (p = 0.018). There was a weak positive correlation between height and the frequency of high-intensity physical activity (r = 0.153, p = 0.022).

Conclusion

This study highlights the complex interactions between sociodemographic, health-related, and environmental factors that influence physical activity in Jazan. The findings underscore the need for targeted, culturally sensitive interventions to promote physical activity, particularly among women, individuals with obesity, and those with chronic conditions. Urban planning should consider the impact of the built environment on physical activity. Future research should use objective measurements and longitudinal methodologies to better understand these relationships and design effective public health interventions in line with Saudi Vision 2030.

## Introduction

Every physiological movement induced by skeletal muscles, including energy expenditure, is considered a type of physical activity [1]. Engaging in physical activity is vital for maintaining health and preventing the development of chronic diseases [1]. Regular physical exercise has been shown to reduce the risk of several illnesses, such as cardiovascular disease, stroke, type 2 diabetes, colon cancer, breast cancer, depression, cognitive decline, and falls [2,3]. The World Health Organization (WHO) recommends that individuals aged 18-64 engage in at least 150 minutes of moderate-intensity aerobic exercise, 75 minutes of high-intensity activity, or a mix of both each week [3,4]. Additionally, they should engage in muscle-strengthening exercises at least two days per week [4].

Inactivity is a significant health problem worldwide, given the many benefits of physical exercise [5]. Approximately 31% of the global population is insufficiently physically active, though inactivity rates vary significantly between countries and regions [6]. Research suggests that lack of physical exercise contributes to 6-10% of the overall disease burden associated with coronary heart disease, type 2 diabetes, breast cancer, and colon cancer [7]. In 2008, inadequate levels of physical activity or exercise were responsible for more than 5.3 million of the 57 million deaths worldwide, accounting for roughly 9% of all premature deaths [7].

In Saudi Arabia, sedentary lifestyles have become increasingly common due to several factors, including rapid economic growth, urbanization, and societal changes [8,9]. Studies indicate that a large proportion of Saudi Arabian children and adults do not engage in any form of physical activity [8,9]. The prevalence of physical inactivity in Saudi Arabia varies across different age groups and geographical locations, ranging from 43.3% to 99.5% [10]. According to the Saudi Health Interview Poll, 66% of the Saudi population, including 61% of men and 72% of women, are physically inactive [11].

Several hurdles hinder the Saudi population from engaging in physical exercise. Time constraints, limited access to suitable exercise facilities, cultural issues, health concerns, insufficient personal motivation, and lack of social support are all barriers to physical activity [12]. Environmental factors also significantly reduce physical activity levels [13], including urbanization, high temperatures, and reliance on vehicles for transportation. Women face additional challenges, such as limited access to facilities and cultural norms, which restrict their ability to participate in physical activity [14]. These barriers contribute to sex-based disparities in physical activity [14,15].

A study conducted among university students in Southern Saudi Arabia revealed a notable prevalence of physical inactivity. Research findings indicated that 91.7% of women and 83.3% of men are inactive [15]. However, there is a shortage of information on physical inactivity among Jazan's adult population and the obstacles preventing them from engaging in physical activity. This research aimed to close that gap by analyzing the prevalence of physical inactivity among Jazan's adult population and identifying the barriers to their participation in physical exercise using validated questionnaires. The findings provide a better understanding of the magnitude of the problem and the factors influencing people's behaviors toward physical exercise. The results can support the creation of targeted initiatives and policies designed to encourage a more active lifestyle. This study contributes to Jazan's long-term public health efforts by promoting physical activity and reducing the prevalence of chronic illnesses.

## Materials and methods

Study design and setting

This study employed a descriptive cross-sectional design and was conducted in Jazan, a city in the southwestern region of Saudi Arabia. Jazan is bordered by the Asir region to the north and east, the Red Sea to the west, and Yemen to the south and east. It has no terrestrial limits.

Population and sampling analysis

This study aimed to gather data from adult residents of the Jazan region. The sample size was determined using a 95% confidence level, a 5% margin of error, and a 10% projected non-response rate. The final sample size was 423 participants. A stratified sampling strategy, consisting of two phases, was employed. In the first phase, the Jazan region was divided into rural and urban strata. In the second phase, three health facilities were randomly selected from each stratum, resulting in the selection of six centers. Study participants were sequentially contacted at each location.

Survey instrument

Data were collected using a self-administered questionnaire with two main components. The first section gathered socio-demographic data, including age, gender, nationality (Saudi or non-Saudi), marital status (married, single, divorced, widowed), number of family members, residence location (city or village), education level (uneducated, middle school or lower, secondary, university), occupation (employed, student, unemployed), monthly income (less than 5000 SAR, 5000-10000 SAR, more than 10000 SAR), and chronic diseases (obesity, hypertension, diabetes).

The second section assessed physical activity levels using the official Arabic short form of the International Physical Activity Questionnaire (IPAQ). The IPAQ brief version includes seven questions that evaluate the frequency and duration of activities categorized as vigorous-intensity, moderate-intensity, walking, and sitting over the previous seven days. Vigorous activities include heavy lifting, digging, aerobics, and fast cycling, which require significant physical effort and substantially increase breathing. Moderate activities, such as carrying light weights, steady cycling, and playing doubles tennis, cause a moderate increase in breathing. Walking encompasses activities like walking for work, transportation, pleasure, or around the home. Sedentary behavior was measured by assessing sitting time on weekdays, including during work, study, and leisure. The validity of the IPAQ has been established, with good measurement characteristics across various scenarios.

Data collection procedure

Trained data collectors contacted individuals at primary health clinics and informed them about the study. The self-administered questionnaire was emailed to participants who volunteered for the study and provided informed consent. Data collectors were available to assist with any questions or concerns. Completed questionnaires were collected on the same day.

Statistical analysis

All statistical analyses were performed using IBM SPSS (IBM SPSS Statistics for Windows, IBM Corp., Version 27.0.1, Armonk, NY). Descriptive and inferential statistical methods were used. Descriptive statistics summarized and characterized participants' attributes and responses, including the frequencies and percentages of categorical variables. The means, standard deviations, medians, and interquartile ranges of continuous variables were calculated and presented. Physical activity levels were compared across different demographic groups using the Mann-Whitney U or Kruskal-Wallis tests. Spearman’s rank correlation test was used to determine the strength of the relationship between continuous variables and physical activity. As the Shapiro-Wilk test (p < 0.05) indicated a non-normal distribution, non-parametric tests were used. All statistical tests used a 95% confidence interval, with a significance threshold of p < 0.05.

Ethical considerations

The research methodology was reviewed and approved by the Research Ethics Committee of Jazan University (permit number 23138). The study adhered to the ethical guidelines outlined in the Declaration of Helsinki. Before data collection, each participant was provided a written informed consent form explaining the study’s objectives, methods, potential risks, and benefits. Participants were informed of their voluntary participation and their right to withdraw from the study at any time without consequence. It was emphasized that their decision to participate or not would not affect the healthcare services they received at the primary health clinics. Only those who provided written informed consent were included in the study. To ensure anonymity, each participant was assigned a unique identification number, and no personal information was collected. Completed questionnaires were securely stored in a restricted area accessible only to the researcher, and electronic data were encrypted and password-protected before storage.

## Results

The study involved 387 participants, with a mean age of 34.0 ± 10.1 years. The gender distribution was 39.0% female and 61.0% male. Most participants were Saudi nationals (98.4%), while a small minority were non-Saudis (1.6%). The average weight and height were 71.3 ± 30.1 kg and 163.2 ± 10.1 cm, respectively. Slightly more participants resided in cities (51.2%) than in villages (48.8%). Regarding marital status, 63.0% were married, 34.6% were single, 2.1% were divorced, and 0.3% were widowed. The mean number of family members was 5 ± 3. In terms of education, 69.8% had a university degree, 25.6% had completed secondary education, 3.4% had middle school or lower education, and 1.3% were uneducated. Employment-wise, 63.8% were employed, 17.3% were students, and 18.9% were unemployed. Monthly income varied, with 37.5% earning less than 5000 SAR, 32.0% earning between 5000 and 10000 SAR, and 30.5% earning more than 10000 SAR. In terms of health conditions, 8.3% of the participants were obese, 6.7% had hypertension, 6.5% had diabetes, and 6.2% had asthma. Regarding smoking, 73.9% were non-smokers, 20.7% were smokers, and 5.4% were ex-smokers. Proximity to recreational facilities showed that 42.6% lived 1-5 km away from the nearest walkway or sports area, 28.9% lived less than 1 km away, and 28.4% lived more than 5 km away (Table 1).

**Table 1:** Sociodemographic and clinical characteristics of participants in the study (N = 387)

Sociodemographic and clinical characteristics		N / mean	(%) / SD
Age		34.0	10.1
Gender	Female	151	(39.0%)
	Male	236	(61.0%)
Nationality	Non-Saudi	6	(1.6%)
	Saudi	381	(98.4%)
Weight		71.3	30.1
Height		163.2	10.1
Residence	City	198	(51.2%)
	Village	189	(48.8%)
Marital status	Divorced	8	(2.1%)
	Married	244	(63.0%)
	Single	134	(34.6%)
	Widowed	1	(0.3%)
Number of family members		5	3
Education	Middle or less	13	(3.4%)
	Secondary	99	(25.6%)
	Uneducated	5	(1.3%)
	University	270	(69.8%)
Occupation	Employed	247	(63.8%)
	Student	67	(17.3%)
	Unemployed	73	(18.9%)
Monthly income	5000 to 10000	124	(32.0%)
	Less than 5000	145	(37.5%)
	More than 10000	118	(30.5%)
Obesity		32	(8.3%)
Hypertension		26	(6.7%)
Diabetes		25	(6.5%)
Asthma		24	(6.2%)
Smoking status	Ex-smoker	21	(5.4%)
	Non-smoker	286	(73.9%)
	Smoker	80	(20.7%)
How far is the nearest walkway or sports area from your home?	1-5 km	165	(42.6%)
	Less than 1 km	112	(28.9%)
	More than 5 km	110	(28.4%)

Over the past seven days, participants exhibited varying levels of physical activity. On average, they engaged in high-intensity physical activity for 3.2 days (SD = 1.7), with a median of 3.0 days (IQR = 2.0-5.0). Notably, 42.1% (163 individuals) did not participate in high-intensity physical activity. Among those who did, the average duration was 74.2 minutes per day (SD = 50.7), with a median of 60.0 minutes (IQR = 35.0-120.0). Moderate-intensity physical activity was undertaken for an average of 3.3 days (SD = 1.7), with a median of 3.0 days (IQR = 2.0-5.0), and 39.8% (154 participants) did not engage in any. On active days, the average duration of moderate-intensity activity was 69.5 minutes (SD = 50.0), with a median of 60.0 minutes (IQR = 30.0-90.0). Walking for at least 10 minutes occurred on average for 4.5 days (SD = 2.0), with a median of 5.0 days (IQR = 3.0-7.0), although 26.6% (103 participants) did not engage in walking. On walking days, participants spent an average of 67.5 minutes (SD = 58.9), with a median of 60.0 minutes (IQR = 30.0-60.0). Additionally, participants spent an average of 260.7 minutes sitting on non-weekend days (SD = 171.3), with a median of 240.0 minutes (IQR = 120.0-360.0) (Table 2).

**Table 2:** Physical activity of participants

Physical activity of participants	Mean ± SD	Median (IQR)
How many days did you engage in high-intensity physical activity during the past seven days?	3.2 ± 1.7	3.0 (2.0-5.0)
	163 participants (42.1%) participants did not do any high-intensity physical activity	
Typically, how much time (minutes) did you spend doing vigorous physical activity on one of those days?	74.2 ± 50.7	60.0 (35.0-120.0)
How many days did you engage in moderate-intensity physical activity during the past seven days?	3.3 ± 1.7	3.0 (2.0-5.0)
	154 participants (39.8%) did not do any moderate-intensity physical activity	
Typically, how much time (minutes) did you spend doing moderate-intensity physical activity on one of those days?	69.5 ± 50.0	60.0 (30.0-90.0)
During the past seven days, how many days did you walk for at least 10 minutes each time?	4.5 ± 2.0	5.0 (3.0-7.0)
	103 participants (26.6%) did not engage in walking	
Typically, how much time (minutes) did you spend walking on one of those days?	67.5 ± 58.9	60.0 (30.0-60.0)
During the past seven days, how much time did you spend sitting on any of these days other than the weekend?	260.7 ± 171.3	240.0 (120.0-360.0)

Analyzing factors influencing engagement in high-intensity physical activity revealed several statistically significant associations. Participants with a monthly income of 5000-10000 SAR reported more frequent high-intensity physical activity, with a median of 3.0 days (IQR = 2.0-5.0), compared to those with higher incomes (more than 10000 SAR), who had a median of 3.0 days (IQR = 1.0-4.0). Obesity was also significantly associated; participants without obesity reported more frequent high-intensity activity (median = 3.0 days, IQR = 2.0-5.0) compared to those with obesity (median = 2.0 days, IQR = 1.0-3.0). Similarly, participants without diabetes engaged more frequently in high-intensity activity (median = 3.0 days, IQR = 2.0-5.0) compared to those with diabetes (median = 2.0 days, IQR = 1.0-3.0). These findings highlight income level, obesity, and diabetes status as significant determinants of high-intensity physical activity. No other factors were significantly associated (Table 3).

**Table 3:** Comparison of high-intensity physical activity among various categorical factors IQR: interquartile range *p < 0.05, significant

Factors		How many days did you engage in high-intensity physical activity during the past seven days?		p-value
		Median	IQR	
Gender	Female	3.0	(2.0-4.0)	0.891
	Male	3.0	(2.0-5.0)	
Nationality	Non-Saudi	1.0	(1.0-5.0)	0.315
	Saudi	3.0	(2.0-5.0)	
Residence	City	3.0	(2.0-4.0)	0.607
	village	3.0	(2.0-5.0)	
Marital status	Divorced	4.0	(2.0-5.5)	0.119
	Married	3.0	(2.0-4.0)	
	Single	3.0	(2.0-5.0)	
	Widowed	1.0	(1.0-1.0)	
Education	Middle or less	2.0	(2.0-3.0)	0.405
	Secondary	2.0	(2.0-5.0)	
	Uneducated	3.0	(1.0-5.0)	
	University	3.0	(2.0-5.0)	
Occupation	Employed	3.0	(2.0-5.0)	0.822
	Student	3.0	(2.0-4.0)	
	Unemployed	3.0	(2.0-4.0)	
Monthly income	5000 to 10000	3.0	(2.0-5.0)	0.014*
	Less than 5000	3.0	(2.0-4.0)	
	More than 10000	3.0	(1.0-4.0)	
Obesity	Yes	2.0	(1.0-3.0)	0.039*
	No	3.0	(2.0-5.0)	
Hypertension	Yes	2.0	(2.0-3.0)	0.057
	No	3.0	(2.0-5.0)	
Diabetes	Yes	2.0	(1.0-3.0)	0.016*
	No	3.0	(2.0-5.0)	
Asthma	Yes	3.0	(2.0-5.0)	0.879
	No	3.0	(2.0-5.0)	
Smoking status	Ex-smoker	3.0	(2.5-5.0)	0.351
	Non-smoker	3.0	(2.0-4.0)	
	Smoker	2.5	(2.0-4.0)	
How far is the nearest walkway or sports area from your home?	1-5 km	3.0	(2.0-4.0)	0.737
	Less than 1 km	3.0	(2.0-5.0)	
	More than 5 km	3.0	(2.0-5.0)	

Regarding moderate-intensity physical activity, proximity to walkways or sports areas showed a borderline significant relationship. Participants living 1-5 km away reported a higher median of 3.0 days (IQR = 2.0-5.0) compared to those living less than 1 km away, who had a median of 2.0 days (IQR = 2.0-4.0). Other factors, including sex, nationality, residence type, marital status, education level, occupation, monthly income, obesity, hypertension, diabetes, asthma, and smoking status, were not significantly associated with moderate-intensity physical activity (Table 4).

**Table 4:** Comparison of moderate-intensity physical activity among various categorical factors IQR: interquartile range *p < 0.05, significant

Factors		How many days did you engage in moderate-intensity physical activity during the past seven days?		p-value
		Median	IQR	
Gender	Female	3.0	(2.0-5.0)	0.804
	Male	3.0	(2.0-5.0)	
Nationality	Non-Saudi	4.0	(2.0-6.0)	0.530
	Saudi	3.0	(2.0-5.0)	
Residence	City	3.0	(2.0-4.0)	0.798
	village	3.0	(2.0-5.0)	
Marital status	Divorced	2.0	(1.0-4.0)	0.429
	Married	3.0	(2.0-5.0)	
	Single	3.0	(2.0-5.0)	
	Widowed	-	-	
Education	Middle or less	2.0	(1.0-5.0)	0.590
	Secondary	3.0	(2.0-5.0)	
	Uneducated	-	-	
	University	3.0	(2.0-4.5)	
Occupation	Employed	3.0	(2.0-4.0)	0.239
	Student	3.0	(2.0-4.0)	
	Unemployed	4.0	(2.0-5.0)	
Monthly income	5000 to 10000	3.0	(2.0-5.0)	0.545
	Less than 5000	3.0	(2.0-5.0)	
	More than 10000	3.0	(2.0-4.0)	
Obesity	Yes	3.0	(1.0-3.0)	0.238
	No	3.0	(2.0-5.0)	
Hypertension	Yes	3.0	(2.0-5.0)	0.735
	No	3.0	(2.0-5.0)	
Diabetes	Yes	3.5	(3.0-5.0)	0.229
	No	3.0	(2.0-5.0)	
Asthma	Yes	2.5	(1.0-4.0)	0.092
	No	3.0	(2.0-5.0)	
Smoking status	Ex-smoker	4.0	(3.0-5.0)	0.183
	Non-smoker	3.0	(2.0-5.0)	
	Smoker	3.0	(2.0-4.0)	
How far is the nearest walkway or sports area from your home?	1-5 km	3.0	(2.0-5.0)	0.05
	Less than 1 km	2.0	(2.0-4.0)	
	More than 5 km	3.0	(2.0-5.0)	

Analyzing factors associated with walking for at least 10 minutes over the past seven days, statistically significant associations were observed for gender and diabetes status. Male participants reported a higher median of 5.0 days (IQR = 3.0-7.0) compared to females, who reported a median of 4.0 days (IQR = 3.0-6.0). Regarding health, participants without diabetes had a median of 5.0 days (IQR = 3.0-6.0), while those with diabetes reported a higher median of 6.0 days (IQR = 5.0-7.0). Other factors, including nationality, residence type, marital status, education level, occupation, monthly income, obesity, hypertension, asthma, smoking status, and proximity to recreational facilities, were not significantly associated with walking frequency (Table 5).

**Table 5:** Comparison of walking among various categorical factors IQR: interquartile range *p < 0.05, significant

Factors		During the past seven days, how many days did you walk for at least 10 minutes each time?		p-value
		Median	IQR	
Gender	Female	4.0	(3.0-6.0)	0.033*
	Male	5.0	(3.0-7.0)	
Nationality	Non-Saudi	5.0	(5.0-7.0)	0.415
	Saudi	5.0	(3.0-7.0)	
Residence	City	4.5	(3.0-6.0)	0.172
	village	5.0	(3.0-7.0)	
Marital status	Divorced	5.0	(4.0-5.0)	0.059
	Married	5.0	(3.0-6.0)	
	Single	5.0	(4.0-7.0)	
	Widowed	-	-	
Education	Middle or less	5.0	(2.0-7.0)	0.408
	Secondary	5.0	(3.0-7.0)	
	Uneducated	1.0	(1.0-1.0)	
	University	5.0	(3.0-6.0)	
Occupation	Employed	5.0	(3.0-6.0)	0.339
	Student	5.0	(3.0-7.0)	
	Unemployed	5.0	(3.0-7.0)	
Monthly income	5000 to 10000	5.0	(3.0-7.0)	0.853
	Less than 5000	5.0	(3.0-6.0)	
	More than 10000	5.0	(3.0-6.0)	
Obesity	Yes	5.0	(3.0-7.0)	0.729
	No	5.0	(3.0-7.0)	
Hypertension	Yes	4.0	(3.0-7.0)	0.923
	No	5.0	(3.0-7.0)	
Diabetes	Yes	6.0	(5.0-7.0)	0.018*
	No	5.0	(3.0-6.0)	
Asthma	Yes	5.0	(1.0-7.0)	0.819
	No	5.0	(3.0-7.0)	
Smoking status	Ex-smoker	5.5	(5.0-7.0)	0.148
	Non-smoker	5.0	(3.0-7.0)	
	Smoker	5.0	(3.0-5.0)	
How far is the nearest walkway or sports area from your home?	1-5 km	5.0	(3.0-7.0)	0.594
	Less than 1 km	5.0	(3.0-6.0)	
	More than 5 km	4.0	(3.0-6.0)	

Height was found to have a weak positive correlation with the frequency of high-intensity physical activity (r = 0.153, p = 0.022), indicating that taller individuals tended to engage in more frequent high-intensity activity. No statistically significant correlations were observed for age, weight, or number of family members in relation to physical activity levels (Table 6).

**Table 6:** Correlation of continuous factors and physical activity *p < 0.05, significant

Factors		How many days did you engage in high-intensity physical activity during the past seven days?	How many days did you engage in moderate-intensity physical activity during the past seven days?	During the past seven days, how many days did you walk for at least 10 minutes each time?
Age	Correlation coefficient	-0.094	0.047	0.006
	p-value	0.161	0.471	0.916
Weight	Correlation coefficient	-0.068	-0.015	0.016
	p-value	0.314	0.817	0.788
Height	Correlation coefficient	0.153	0.076	0.113
	p-value	0.022*	0.245	0.056
Number of family members	Correlation coefficient	-0.070	0.081	0.103
	p-value	0.296	0.221	0.084

## Discussion

This study provides important insights into physical activity patterns and associated factors among the adult population in the Jazan region of Saudi Arabia. These findings highlight key aspects of physical activity participation and identify potential areas for intervention and further research.

High-intensity physical activity

Our data suggest that income, obesity, and diabetes status influence participation in high-intensity physical activity. Notably, individuals with monthly incomes ranging from 5000 to 10000 SAR reported engaging in high-intensity physical activity more frequently than those with higher incomes. These findings contradict previous studies that found a positive relationship between income and physical activity levels [16]. However, they align with research indicating that the relationship between wealth and physical activity is complex and may vary across different populations and contexts [17].

The lower engagement in vigorous physical activity among higher-income participants in our study might be due to longer working hours, sedentary jobs, or increased reliance on motorized transportation. This finding is consistent with studies conducted in other Gulf Cooperation Council (GCC) countries, which have linked fast economic growth to more sedentary lifestyles [18]. In Qatar, a high-income Gulf country, Al-Kuwari et al. (2016) found that people with higher socioeconomic status, especially women, engaged in less physical activity [19].

Previous studies have repeatedly shown a detrimental relationship between obesity and high-intensity physical activity [20]. Obesity appears to be both a cause and a consequence of reduced physical activity. Myers et al. (2017) provided convincing evidence of the reciprocal connection between physical activity and obesity, highlighting the need for public health campaigns to address both issues simultaneously [20]. Individuals with diabetes were less likely to participate in high-intensity physical activity, consistent with prior findings [21]. This emphasizes the importance of promoting safe and appropriate physical activity among individuals with chronic conditions. Colberg et al. (2016) stressed the need for personalized physical activity plans for people with diabetes, given the challenges they may face, such as fear of hypoglycemia or lack of awareness of safe exercise routines [22].

Moderate-intensity physical activity

Our research revealed a significant relationship between proximity to walkways or sports facilities and engagement in moderate-intensity physical activity. This finding highlights the role of the environment in promoting physical activity, as shown in previous studies [23]. Interestingly, participants who lived 1-5 km away from these facilities engaged in more moderate-intensity activity compared to those living closer, a finding that warrants further investigation.

This result contrasts with earlier studies that reported a positive relationship between proximity to recreational facilities and physical activity levels [24]. However, it aligns with the "optimal location" concept in urban planning, where a certain distance from facilities may encourage intentional physical activity. Althoff et al. (2017) demonstrated that the physical environment significantly influences physical activity levels using smartphone data [25]. However, this relationship is not always straightforward and may vary based on other factors.

Walking behavior

The observed gender differences in walking activity, with men reporting more frequent walking than women, are consistent with previous studies conducted in Saudi Arabia and other Gulf countries [26]. This disparity may be due to cultural norms, safety concerns, or differences in daily routines and responsibilities. Al-Eisa and Al-Sobayel (2012) noted that Saudi women face unique challenges regarding physical activity, such as time constraints, lack of facilities, and social support [14]. Our findings highlight the persistence of gender disparities and the need for tailored interventions for both sexes.

The higher incidence of walking among participants with diabetes is encouraging, as it suggests that they may be adopting walking as a way to regulate their physical activity. This aligns with diabetes management guidelines that recommend lifestyle modifications [22]. According to Qiu et al. (2014), walking is the most popular form of physical activity among people with diabetes due to its accessibility and ease [27].

Correlation with height

We found a modest positive relationship between height and frequency of high-intensity physical activity. Although limited research has explored this relationship, some studies have shown correlations between height and participation in specific types of physical activity or sports [28]. Factors such as body composition, physical strength, and cultural perceptions may influence these associations.

Tyrrell et al. (2016) used Mendelian randomization to identify a causal link between height and participation in certain physical activities, particularly those that benefit from longer limbs [28]. However, the authors acknowledged that this relationship is complex and influenced by various biological and social factors. Our findings add to the existing body of research and highlight the need for further investigation into the impact of anthropometric factors on physical activity patterns.

Sociodemographic factors and physical activity

Our study did not find a statistically significant relationship between education level and physical activity, which contrasts with numerous previous studies. Alqahtani et al. found a clear link between education and physical activity levels in Saudi Arabia [29]. The lack of association in our study may be due to the relatively homogeneous educational background of our sample or other regional characteristics specific to Jazan. Similarly, we did not observe a significant association between age and physical activity, which deviates from the global trend of declining physical activity with age [30]. This could be attributed to cultural factors specific to the Jazan region or Saudi Arabia, where traditional practices or family obligations may promote physical activity across all age groups.

Limitations and future directions

It is important to acknowledge the limitations of this study. First, relying on self-reported physical activity data may introduce recall and social desirability biases. Additionally, the cross-sectional design of this study limits our ability to establish causal relationships. Longitudinal studies are needed to confirm the factors influencing physical activity patterns in Saudi Arabia. Moreover, while our findings provide valuable insights into physical activity in the Jazan region, they may not be representative of the entire Saudi population. Future research should focus on conducting large, nationally representative surveys to better understand physical activity patterns across Saudi Arabia.

Implications for public health

The findings of this study have significant implications for public health initiatives in Saudi Arabia. Targeted programs should be implemented to promote physical activity among specific populations, such as women, individuals with obesity, and those with chronic diseases like diabetes. Furthermore, the role of environmental factors in encouraging physical activity underscores the need for urban planning policies that prioritize the development of accessible recreational facilities and walkways. This aligns with Saudi Vision 2030, which emphasizes promoting a healthy lifestyle among residents [31].

The complex relationship between socioeconomic factors and physical activity also highlights the need for comprehensive health promotion efforts that address the diverse needs of different socioeconomic groups. Workplace interventions for higher-income individuals and community-based programs for lower-income groups, as suggested by Heath et al. (2012), may help promote physical activity [32].

This study provides valuable insights into physical activity patterns and influencing factors in the Jazan region of Saudi Arabia. These findings contribute to the growing body of knowledge on physical activity in the Middle East and offer important recommendations for future research and public health initiatives aimed at promoting active lifestyles and reducing noncommunicable diseases in Saudi Arabia. Given the nation’s rapid socioeconomic changes, it is essential to continuously evaluate and adapt interventions to meet the evolving physical activity needs of the population.

## Conclusions

This study provides important insights into the patterns of physical activity and the factors that influence it among individuals in the Jazan region of Saudi Arabia. The findings of our research reveal complex relationships between sociodemographic, health-related, and environmental factors and physical activity levels. Key findings include the effects of income level, obesity, and diabetes status on high-intensity physical activity, the importance of the built environment for moderate-intensity activity, the persistence of sex differences in walking behavior, and a positive relationship between height and the frequency of high-intensity activity.

The implications of these findings for Saudi public health initiatives are significant, suggesting the need for targeted, culturally relevant interventions, as well as the incorporation of environmental factors into urban development programs. The distinguishing features of this study are its focus on a specific region and a detailed analysis. However, several limitations should be noted, including reliance on self-reported data and the use of a cross-sectional design. Future research should employ objective measures and longitudinal methodologies to clarify causal links and explore cultural influences. This study contributes to the existing body of knowledge on physical activity in Saudi Arabia and the Middle East. It serves as a foundation for adopting evidence-based strategies to promote physical activity and combat non-communicable diseases, aligning with the health-related goals of Vision 2030.
